# Pioneers of cortical cytoarchitectonics: the forgotten contribution of Herbert Major

**DOI:** 10.1007/s00429-024-02825-0

**Published:** 2024-07-02

**Authors:** Andrew J. Larner, Lazaros C. Triarhou

**Affiliations:** 1 Department of Brain Repair and Rehabilitation, Institute of Neurology, University College London, London, UK; 2 Department of Psychology, Division of Brain, Behavior and Cognition, Aristotelian University Faculty of Philosophy, Thessaloníki, Greece

**Keywords:** Betz, Cerebral cortex, Cortical layers, Cytoarchitectonics, History of neuroscience

## Abstract

The study of cortical cytoarchitectonics and the histology of the human cerebral cortex was pursued by many investigators in the second half of the nineteenth century, such as Jacob Lockhart Clarke, Theodor Meynert, and Vladimir Betz. Another of these pioneers, whose name has largely been lost to posterity, is considered here: Herbert Coddington Major (1850–1921). Working at the West Riding Asylum in Wakefield, United Kingdom, Major’s thesis of 1875 described and illustrated six-layered cortical structure in both non-human primates and man, as well as “giant nerve cells” which corresponded to those cells previously described, but not illustrated, by Betz. Further journal publications by Major in 1876 and 1877 confirmed his finding of six cortical strata. However, Major’s work was almost entirely neglected by his contemporaries, including his colleague and sometime pupil at the West Riding Asylum, William Bevan-Lewis (1847–1929), who later (1878) reported the presence of both pentalaminar and hexalaminar cortices. Bevan-Lewis’s work was also later credited with the first illustration of Betz cells.

## Introduction

The history of the study of cortical cytoarchitectonics dates back to the late eighteenth century and the independent findings of Francesco Gennari, Felix Vicq d’Azyr, and Samuel von Soemmering in the 1780s of a myelinated band in the occipital cortex. With the advent from the mid-nineteenth century onwards of new techniques in brain sectioning and staining which permitted microscopical examination of cortical tissue, various descriptions of the laminar structure of cortex appeared. However, consensus as to the six-layered nature of mammalian neocortex was slow to emerge, with various opinions expressed as to the exact number of strata, ranging through four (Kölliker 1854), five (Meynert 1872), six (Baillarger 1840), eight (Clarke 1862–1863), and even nine layers (Ramón y Cajal 1899; DeFelipe and Jones 1988).

Studies of brain histology also addressed the various cell types seen within the cortex. In 1874 Vladimir Betz had described, but not illustrated, giant pyramidal cells in the precentral gyrus. Initially published in Russian, his work was later translated into German in 1874 and English in 1875 (Betz 1875). The comparative histology of the brains of human and non-human primates was also topical at this period in the last quarter of the nineteenth century. For example, Thomas Huxley had written a “Note on the resemblances and differences in the structure and the development of the brain in man and apes” for the second edition of Darwin’s *Descent of man* published in 1874 (Darwin 1874).

Of the many researchers contributing to these studies of cortical histology (Hakosalo 2006; Triarhou 2020, 2021), one who remains known to posterity is William Bevan-Lewis (1847–1929). Working at the West Riding Asylum at Wakefield in the north of England, where David Ferrier had undertaken experimental studies in 1873 which characterised the motor areas of the cerebral cortex (Larner 2023a), Bevan-Lewis’s publications in 1878 described a five-layered cortex in the motor area (Lewis and Clarke 1878; Lewis 1878), as per the influential model of Theodor Meynert. Bevan-Lewis also illustrated, apparently for the first time, the giant cells of the motor cortex previously described by Vladimir Betz (1874).

However notable the studies of Bevan-Lewis were, it transpires that he was not the first pathologist from the West Riding Asylum at Wakefield to investigate, describe and illustrate the strata of the cortex, or comment on and illustrate giant nerve cells. His predecessor, and in some senses his mentor, Herbert Coddington Major (1850–1921), had published on and illustrated six cortical layers in the human and non-human primate brain in his doctoral thesis of 1875 (Major 1875a) and in papers published in 1876 and 1877 (Major 1875-1876a, 1876, 1877a), in contrast to Bevan-Lewis’s initial view of a five-layered cortical organisation in the motor area. Moreover, in his 1875 thesis Major described and illustrated giant nerve cells in the cortical layers.

### Herbert Coddington Major (1850–1921)

As, to our knowledge, only one brief biographical article on Major has appeared (Larner 2024), some details of his life are given before proceeding to discuss his work in cortical cytoarchitectonics and histology.

Born in Jersey, in the Channel Islands, on 30th January 1850, he was baptised Herbert Coddington Mauger (pronounced Major). After his school education in Jersey, he went to Edinburgh to study medicine and graduated (MB CM) in 1871. During his time in Edinburgh he attended the class in Medical Psychology set up by Thomas Laycock (1812–1876), Chair of the Practice of Physic at Edinburgh University from 1855. This instruction in medical psychology and mental diseases was novel in British medical schools at this time and served to influence a number of Edinburgh students to take up careers in this discipline, either in asylum medicine (e.g. James Crichton-Browne, Thomas McDowall, Robert Lawson) or in general medicine with an interest in diseases of the brain (David Ferrier, John Milner Fothergill). Prior to his translation to Edinburgh, Laycock had also influenced the young John Hughlings Jackson during his studies at the York Medical School in the 1850s.

By the time Major, as he was now known, graduated from Edinburgh, James Crichton-Browne had been the Medical Superintendent at the West Riding Asylum in Wakefield for some years, setting up the facilities and recruiting the personnel required to undertake systematic studies of patients with insanity. These facilities included a dedicated pathological laboratory. Into this environment, which has latterly been characterised as a “research school” (Finn 2012), Major was introduced as a Clinical Clerk (unpaid, but in receipt of board and lodging) in 1871. Having proved himself, in the words of Crichton-Browne, “an indefatigable Clinical Clerk for twelve months”, Major was “promoted to the position of Assistant Medical Officer, which he now occupies with credit”. This promotion to a salaried position was announced in the medical journals in August 1872.

Major’s interest and meticulous work in pathology was already well underway at this time. At the Annual Meeting of the British Medical Association held in Birmingham in August 1872, Crichton-Browne showed “some beautifully prepared sections of Brain-Structure [*sic*] in Health and Disease, the work of Dr. Herbert C. Major of the West Riding Asylum”. Major attended the medical *conversazione* at the Asylum in October 1872, an annual meeting arranged by Crichton-Browne to showcase the work of the institution, particularly the research projects undertaken by members of the resident junior staff. Major presided over a table “filled with a large number of microscopical preparations from the Asylum collection” (Anon., 1872), no doubt many prepared by Major himself. He repeated this display at the *conversazione* of 1873, 1874, and 1875.

It was in 1872 that Major initiated his series of papers published in the *West Riding Lunatic Asylum Medical Reports*, the house journal of the Asylum which had been founded by Crichton-Browne to disseminate the findings of research undertaken there (Larner 2023b). Over the next four years, Major published six papers in this journal (Major 1872a, b, 1873, 1874a, 1875b, 1876), more than anyone else save Crichton-Browne, as well as elsewhere (Major 1874b, 1875-1876b). He also completed his thesis, *Histology of the brain in apes* (Major used the term “apes” in a manner different from current usage) for the MD degree of Edinburgh University and which received the gold medal (Major 1875a). This interest in comparative neurohistology afforded further publications (Major 1875-1876a, 1877a). It is little wonder then that one of his junior colleagues at Wakefield Asylum, John Hunter Arbuckle, described Major at this time as “the first authority on the minute structure of the cerebral cortex of man and monkeys” (Arbuckle 1876).

In 1875, William Bevan-Lewis was appointed as a Clinical Assistant at the Asylum (Larner and Triarhou 2023) where Major, now the Deputy Medical Director (Major 1874a), encouraged him as he began his career in pathological research.

Asylum life was not all work. Consistent with practice in other asylums of the time, the patients at Wakefield were provided with entertainment in the form of dances, concerts and amateur theatricals, with Asylum staff often taking roles in the latter. Major was no exception, for example appearing in the farce “The Day after the Wedding” on 17th November 1874 in the role of “Colonel Freelove”, and in the comedy “Faint heart never won fair lady” on 12th February 1875 as “GUZMAN (a Gentleman, who by becoming a Page turns over a new leaf, as he usually uses his *High Powers* on more distinguished parts)” [capitals and italics in original playbill; West Yorkshire Archive Service, C85/1362].

Major was appointed Medical Director of the West Riding Asylum in early 1876 following Crichton-Browne’s resignation. The administrative burden of the role undoubtedly took him away from his pathological work, as indicated by the diminution in his published output (Major 1879, 1879–1880, 1882–1883) and a turn towards administrative data (Major 1877b, 1884–1885). He resigned the superintendency in 1884 on the grounds of ill health, to be succeeded by Bevan-Lewis. Major resumed clinical work, as an honorary physician at Bradford Infirmary, in 1885 and became consultant physician in 1898, before moving to Bedford in 1900 as Honorary Pathologist to the Bedford County Hospital. He retired to Jersey in 1907, having married Mary Ann Balleine there in 1906. Major died in 1921, in relative obscurity, having moved in 1920 to Oxford. To our knowledge, only a single obituary was published (Anon., 1921).

### Major’s key publications on cortical cytoarchitectonics

Four of Major’s publications relate specifically to cortical cytoarchitectonics and histology (Major 1875a, 1875-1876a, 1876, 1877a). Each of these will be addressed in turn, but it should first be noted that although Major had certainly written on cortical layers in previous publications, in only two instances was a layer qualified numerically, specifically as the “second layer” (Major 1873: p 102; 1875b: p 168). That he recognised there were more than two cortical layers might also be inferred from his account written in 1872:

In all my sections of the grey matter in this part [occipital lobes] of the healthy brain, I have found the arrangement of the cell elements to be singularly constant. The large nerve cells form two distinct layers, one of which lies superficial, the other on the deep aspect of another well marked intermediate layer, formed almost entirely of small round or oval nerve cells and nuclei. The latter is situate about midway in the depth of the cortical substance. (Major 1872a: pp 49-50).

Nowhere, however, had Major illustrated cortical lamination, his drawings being limited to particular cell types as observed in healthy and diseased brain.

**Fig. 1:**
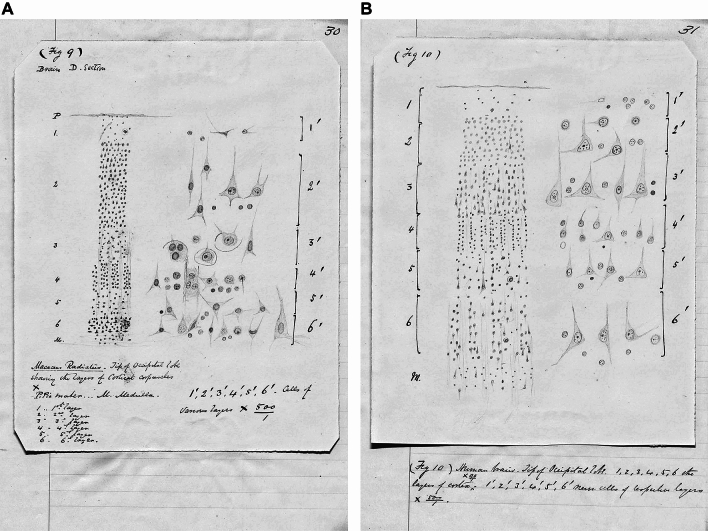
**A** Fig. 9 (p 30) and (**B**) Fig. 10 (p 31) drawings from Major’s thesis (Major 1875a), respectively illustrating the hexalaminar cortex of “ape” (non-human primate) and man

Thus, it was in his thesis, *Histology of the brain in apes*, that Major first described and illustrated the laminar structure of the brain in humans and non-human primates as being comprised of six layers (Major 1875a). This handwritten work was produced “after a period of four years of almost constant study of the human brain” (Major 1875a: p 4; our transcription). Major used two human brains as “standards of comparison”, from men aged 16 and 21 who had both been killed suddenly. The brains from eight different species of non-human primate were available to Major, their sources unspecified. By his own account, the methodology for the preparation of tissues followed that of Lockhart Clarke (Clarke 1862–1863). It was from “Brain D”, of the “*Macacus radiatus*” or Bonnet monkey (now *Macaca radiata*, Bonnet macaque), that the illustration of the cortical layers was made (Major 1875a: p 30, his Fig. 9). This was contrasted with a section from the healthy human brain (Major 1875a: p 31, his Fig. 10) but it was not specified from which human subject it came. These beautiful drawings leave no room for ambiguity about the hexalaminar structure perceived by Major and his text describes the cell types in each layer, along with illustrations thereof (Fig. 1).

**Fig. 2:**
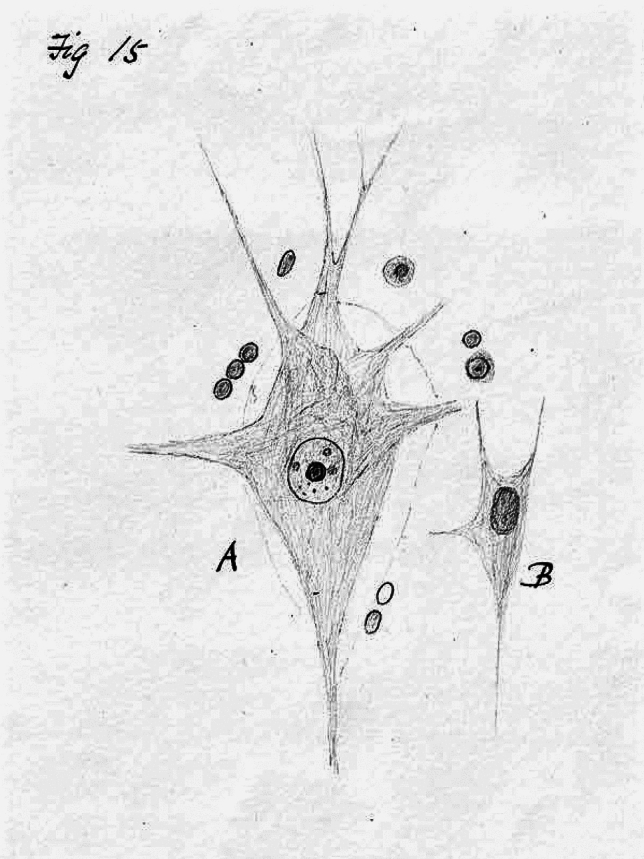
Fig. 15 (p 62) drawing from Major’s thesis, illustrating “giant nerve cells” in the ascending parietal convolution of human brain. Major denoted A as “Large cell” and B as “cell of ordinary size”

In addition to the lamination, Major’s text described the cell types in each layer, along with illustrations thereof (Fig. 1A, B). Towards the end of the thesis, he also mentioned “bodies to which I have given the name of giant nerve cells” (Major 1875a: p 61) which he had observed in the ascending parietal convolution, but neither layer nor cell size was specified. Of these giant nerve cells (Fig. 2), Major commented that “There can be no mistaking them when they have once been seen, their rarity and their great size as compared with the other corpuscles surrounding them at once attracting notice. The branches are very numerous” (Major 1875a: p 63; our transcriptions). No mention was made of the work of Betz. Major had previously noted similar cells in the brain of a patient afflicted with general paralysis (Major 1874a).

In the January 1876 issue of the *Journal of Mental Science*, Major published his histological findings from the brain of a baboon (Major 1875-1876a), called by him a Chacma Baboon or *Cynocephalus porcarius* (now *Papio ursinus*). Prefacing his findings, Major stated that:

My own work in this direction has till now been limited to the brain in the smaller apes, a study of the cortex in which formed the subject of a graduation Thesis presented to the University of Edinburgh. … so far as I have been able to ascertain, this essay was the first record of systematic comparison (though, of course, limited in extent) between the nerve elements of the cortex in man as compared with the ape, and … forms, I believe, at the present time, the only literature of the subject in this or any other country. (1875-1876a: p 500)

With regard to the number and appearance of the cortical strata in the baboon, Major was explicit:

I wish to state at once, and very decidedly: - 1st, that the number in the Chacma corresponds exactly with that in man, in the frontal and parietal, as well as in the occipital lobe (Major 1875-1876a: p 503)

He proceeded to describe and illustrate the cell types in the six layers but, unlike the material in his thesis, there was no drawing here of a section to show the cortical lamination. Writing of the fifth layer, he noted:

In this situation, however, more frequently perhaps than in any other, very large nerve cells are found. Usually, these have the characters of the large nucleated, pale bodies, already frequently referred to, but they sometimes resemble closely the large pyramidal cells before described in connection with the third stratum in the anterior portions of the hemispheres (Major 1875-1876a: p 507)

In the context of the third layer, Major said of these bodies that “wherever seen, their peculiar characters enable them to be recognised at once, so different are they from the others”. Major attempted to measure these pyramidal cells of very large size, finding them “as much as 10/250 mm long by 5/250 mm broad” (Major 1875-1876a: p 505), hence 40 μm by 20 μm.

In the sixth volume of the *West Riding Lunatic Asylum Medical Reports*, dated 1876 but not actually published until early in 1877, Major reported on “The histology of the island of Reil” (Major 1876). Herein, after noting the findings of Kölliker, Lockhart Clarke, and Meynert, he stated that:

In a Thesis presented to the University of Edinburgh (1875), on the ‘Histology of the Brain in Apes,’ I described six cortical layers as being the usual arrangement in the human brain. In the ‘Journal of Mental Science’ for January 1876, in a paper on the brain of the Chacma Baboon, I again showed that in the human subject the six-layer type of the cortex was the usual one. (Major 1876: p 5).

**Fig. 3:**
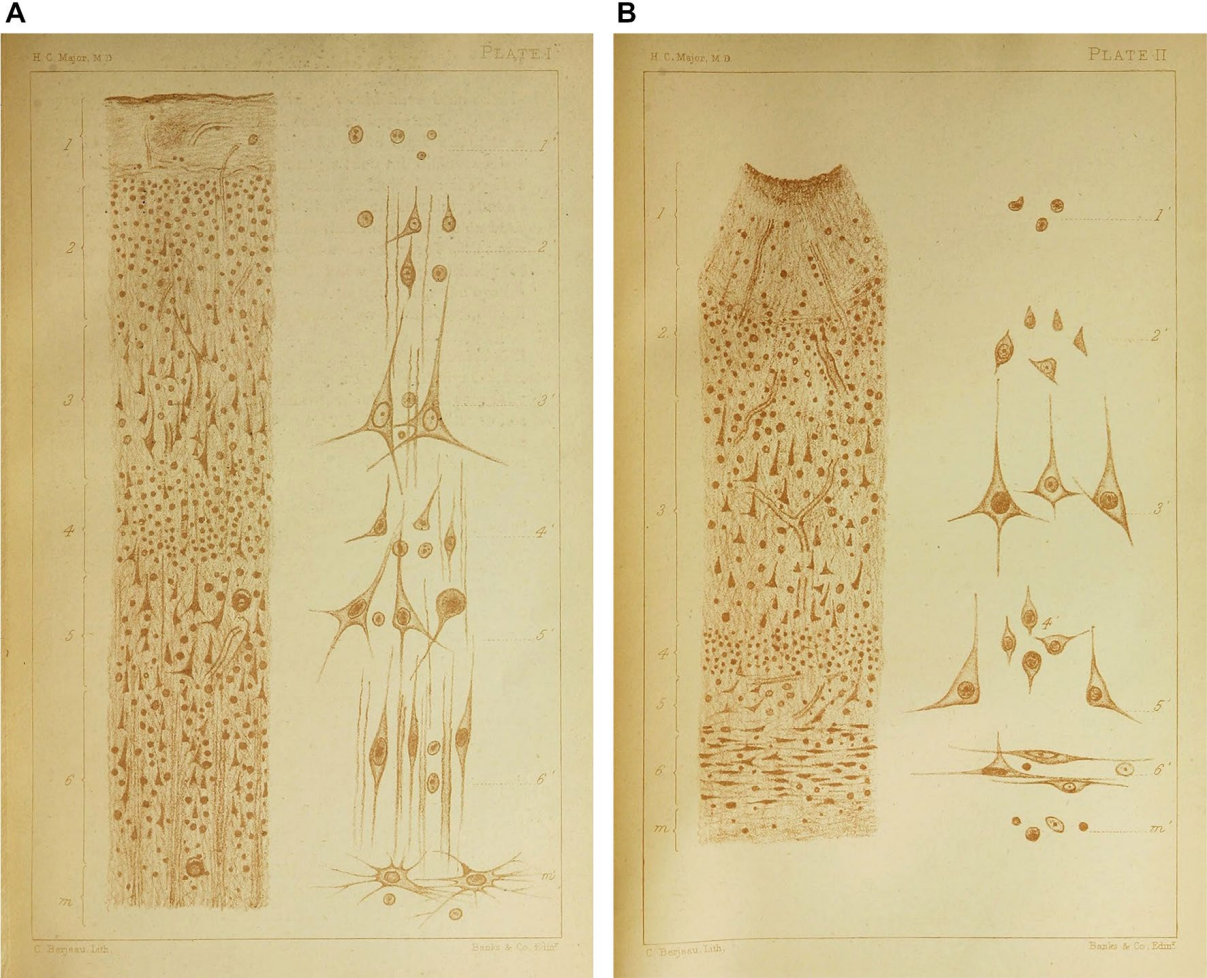
**A** Plate I and (**B**) Plate II from Major 1876, whose explanation of the Plates is as follows: Plate I.—Section through a gyrus of the Island of Reil, showing the cortex of the summit of the gyrus (healthy). Plate II.—Section through the cortex of the Island of Reil at the bottom of a sulcus (morbid). In both:—1, 2, 3, 4, 5, 6, indicate the cortical layers magnified 50 diameters. M M Medulla. 1ʹ, 2ʹ, 3ʹ, 4ʹ, 5ʹ, 6ʹ, cells of the various layers magnified 350 diameters. MʹMʹ Medulla. In the medulla of Plate II corpuscles of Deiter (cellules araignées, Gratiolet) are seen

The hexalaminar cortical appearance was illustrated in this paper with drawings from both healthy and morbid brains, along with the cell types observed (Major 1876: Plate I and Plate II, respectively; reproduced here as Fig. 3). Of the nerve cells, Major commented “I can observe nothing unusual: - nothing that would seem to imply (as in the case of the so-called giant cells of the vertex) any special and peculiar functions” (Major 1876: p 6).

In Major’s two-part *Lancet* paper of July 1877, based on his thesis but using data from only four non-human primate species, rather than the eight reported in the thesis, the six-layered cortex in both human and non-human primates was again illustrated. The drawing of the human brain (Major 1877a: p 46, his Fig. 1) was a reproduction of Plate I from his *West Riding Lunatic Asylum Medical Reports* paper (Major 1876: Plate I; our Fig. 3A). In this paper, Major gave credit for “a human nerve-cell from the cerebral cortex admirably demonstrated by my colleague, Mr. Bevan Lewis, in which the branches are as many as twelve in number” (Major 1877a: p 86). No mention was made of giant nerve cells.

Hence both by description and by illustration, it is clear that Major viewed the cerebral cortex of man and of non-human primates as consisting of six layers, his first illustration of this arrangement dating to his thesis of 1875 and repeated in his papers of 1876 and 1877. He had observed and illustrated “giant nerve cells” in his thesis, but without reference to Betz, and had attempted to measure these cells in the baboon’s brain.

### William Bevan-Lewis (1847–1929)

Herbert Major pre-dated Bevan-Lewis as both pathologist and Medical Superintendent at the West Riding Asylum in Wakefield. Although younger than Bevan-Lewis by three years (Bevan-Lewis came to asylum medicine relatively late in his career), the evidence from Bevan-Lewis’s earliest publications suggests that Major was helpful, if not a mentor, to him. In his first paper, published in the *West Riding Lunatic Asylum Medical Reports*, Bevan-Lewis confirmed the opinion of Major concerning morbid changes in the peripheral nerves of patients with general paralysis (Lewis 1875: p 86), and in a paper on microscopical techniques (a subject Bevan-Lewis was later to make his own) he acknowledged “the valuable assistance and encouragement rendered to me by Dr. Herbert Major – a well-known authority in these matters” (Lewis 1876: p 248).

Furthermore, it is certain that Bevan-Lewis knew of Major’s thesis, since at the annual medical *conversazione* held at Wakefield Asylum in November 1875:

The table presided over by Dr. Herbert Major and Dr. Bevan Lewis was crowded with a quite unique collection of microscopic preparations from the collection belonging to the asylum, illustrating the histological condition of the convolutions of the human brain in the healthy adult, in the foetus, and in various forms of insanity; and similar series of the medulla oblongata, spinal cord, sciatic nerve, and sympathetic ganglia. *Among these preparations were the series illustrating Dr. Major’s thesis for the M.D. degree of Edinburgh, which received the gold medal*, and his own and Dr. Lewis’s papers in the West Riding Reports (Anon., 1875) [our italics].

Bevan-Lewis’s first foray into the subject of cortical architectonics appears to be the paper he co-authored with Henry Clarke which was read at the Royal Society on 24th January 1878, communicated by David Ferrier. In this work describing cortical lamination and the giant cells found in the motor area, only a single mention of Major is to be found, to the effect that Major “follows Baillarger in regarding the cortex of the vault and that of the central lobe as consisting of six layers” (Lewis and Clarke 1878: p. 42). In contrast, Bevan-Lewis and Clarke favoured a five-layered model of the motor area, as illustrated in their Plate 1, following the scheme of Meynert. Their only reference to Major’s papers on the subject was to his 1876 publication in the *West Riding Lunatic Asylum Medical Reports* (Major 1876) but not to his other studies which had described cortical lamination in the brains of human and non-human primates (Major 1875a, 1875-1876a, 1877a).

Bevan-Lewis’s subsequent single-author paper in the inaugural issue of *Brain*, published in April 1878, reported the presence of both pentalaminar and hexalaminar cortices, each typical of a certain definite area, but “no abrupt passage from one form of cortical lamination to that of another is ever seen”. He again referenced Major’s *West Riding Lunatic Asylum Medical Reports* paper to the effect that he “extends the limits of the six-laminated cortex to the central lobe or insula”, but in Bevan-Lewis’s view:

There is a five and a six-laminated cortex, each typical of a certain definite area: but, whilst the six-layered formation is found extensively spread over the convolutions of the parietal and other regions, the *five-laminated* type is pre-eminently characteristic of the motor area of the brain. (Lewis 1878: p 80 [italics in original]).

Bevan-Lewis did refer in passing (Lewis 1878: p 92) to Major’s publication on the Chacma baboon (Major 1875-1876a) although not in relation to cortical lamination, but he did not refer to Major’s other studies which had described cortical lamination in the brains of human and non-human primates (Major 1875a, 1877a). As for cell types, Bevan-Lewis noted of the motor cortex that:

Another highly important feature of this region is the presence of large ganglionic cells which under the title of “giant cells” were made the subject of special attention by Professor Betz over three years ago (Lewis 1878: p 80).

Referencing the German translation of Betz’s original paper, he noted that Betz found these cells to range from 40 to 120 μm long, and from 50 to 60 μm broad, whilst his own measurements were from 30 to 96 μm long, and from 12 to 45 μm broad with a maximum size of 126 by 55 μm. No mention was made of Major’s observation of “giant nerve cells”; admittedly their reported size (40 μm by 20 μm) was somewhat smaller than that found by Betz but within the range reported by Bevan-Lewis.

This mixture of five- and six-layered cortex remained Bevan-Lewis’s position, as evident in the extensive section of 30 pages devoted to cortical lamination in his textbook of mental diseases which first appeared in 1889 (Bevan Lewis 1889: pp 85–114). Here, speaking of the lamination of the motor cortex in man, he stated:

It is all the more essential that its structure in man should be clearly defined here, since it has been the subject of dispute between such writers as Meynert, Betz, Baillarger, Mierzejewski, and others, some authorities speaking of it as a five-laminated and others as a six-laminated type. At the outset, therefore, it is well to define our own view of the case, which is briefly as follows: the cortex typical of motor areas is a five-laminated formation, and the more absolutely the granule cell formation (which, when intercalated, gives us the six-laminated type) is excluded, the more highly specialised become those groups of enormous nerve cells which go by the name of the “nests” of Betz. Where, therefore, these cell-clusters are best represented, there we find a five-laminated, not a six-laminated, cortex; in other words, at these sites the granule-cell layer no longer exists. (Bevan Lewis 1889: p 99).

There is only a single mention of Major in this section, related to his description of cells at the bottom of the fifth layer being “reclinate”, but no reference to Major’s publications is given (Bevan Lewis 1889: pp 101–102).

The absence of Major’s name in the quoted list of writers on the subject of cortical structure is perhaps surprising. Although his studies had principally been on occipital cortex rather than the motor area per se, Lewis and Clarke (1878: p 42) themselves had noted that Major “follows Baillarger in regarding the *cortex of the vault and that of the central lobe as consisting of six layers*” [our italics]. Yet when Bevan-Lewis came to discuss “the six-laminated cortex typical of sensory areas” in his textbook (Bevan Lewis 1889: p 101) there was still no mention of Major. The only two papers by Major cited in the entire textbook (viz. Major 1872, at 471, but without specifying which of his two papers in the *West Riding Lunatic Asylum Medical Reports* of that year; and Major 1879–1880, at 457) do not include his key publications on cortical cytoarchitectonics. Whether these omissions were a mere oversight, astonishing as that possibility now seems, or a wilful exclusion on Bevan-Lewis’s part cannot be decided with the currently available evidence, but a general pattern of minimal or non-citation of his colleague appears to emerge.

## Discussion

Whereas Bevan-Lewis’s “work was fully appreciated by specialists at home and abroad” (Triarhou 2021: p 59), Francis Walshe calling him “the pioneer of cortical cytoarchitectonics” (Walshe 1948: p 208n2), Major’s work was essentially forgotten. True, the Chacma baboon paper was referenced in Henry Maudsley’s *Physiology of mind* (3rd edition) of 1876, but to our knowledge the only discussion of Major’s work on the layers of the cerebral cortex and his differences with Bevan-Lewis appeared in a history of the Wakefield Asylum (Todd and Ashworth, n.d.: pp 162–165) who also acknowledged that “Major never did receive the acclaim that his research in the sphere of comparative neurohistology so richly deserved” (Todd and Ashworth, n.d.: p 178).

The neglect of Major’s meticulous and beautifully illustrated work in cortical cytoarchitectonics has largely persisted for nearly 150 years. He is not mentioned in works on the cerebral cortex by classical authors of his era, including Jules Soury (1899), Ramón y Cajal 1900–1906; DeFelipe and Jones 1988), Theodor Kaes (1907), Otto Marburg (1907), von Economo and Koskinas (1925), Alfons Maria Jakob (1927), Maksymilian Rose (1935), and Cornelius Ariëns Kappers (Ariëns Kappers et al. 1936). Neither is Major mentioned in modern works on the cerebral cortex, such as those by Sarkisov et al. (1949), Bailey and Bonin (1951), Sanides (1962), and Brodal (1981), nor in standard modern texts on the history of neuroscience (Clarke and Jacyna 1987; Finger 1994; Marshall and Magoun 1998).

However, there are some notable exceptions to this neglect of Major’s work. The Swedish physician Carl Hammarberg (1865–1893) completed one of the earliest comprehensive cytoarchitectonic studies of the human cerebral cortex, concentrating both on normal cortical histology and pathology. He wrote his MD thesis in Swedish (Hammarberg 1893), with a title translated as ‘Clinico-pathological studies of intellectual disability along with studies of the normal anatomy of the cerebral cortex’. After his premature death at the age of 28 years, his thesis was translated and published in German (Hammarberg 1895). Hammarberg (1893, 1895) cited Major’s paper on the insula, and mentioned Major on three occasions, as follows (our translations):

Gowers follows Bevan Lewis’ account. Major and Baillarger mention this [3rd] cell layer, but place it between the 4th and 5th layers. (Hammarberg 1895: p 12).

There is probably a confusion here with the authors’ pyramidal cells in (Bevan Lewis) or below (Baillarger, Major) the 4th layer (Hammarberg 1895: p 13).

Obersteiner emphasizes (after H. Major) that the cortex in the insula does not deviate from the common type. (Hammarberg 1895: p 37).

In the textbook on comparative neuroanatomy co-authored by the Polish neurologist Edward Flatau and the Berlin neuroanatomist Louis Jacobsohn published in 1899, two of Major’s articles are cited: on the white whale (Major 1879) and on the Chacma baboon (Major 1875-1876a). Further, Flatau and Jacobsohn (1899: p 110) reported the brain of the orangutan to weigh 375 g, according to Major.

In his landmark monograph on the cerebral cortex, Korbinian Brodmann (1868–1918) cited Major’s 1876 papers on the insula and the Chacma baboon. In a footnote to the opening statement (“Since the first pioneering research of Meynert and Betz, a continuous stream of workers has studied the cellular lamination of the cerebral cortex and its specific modifications in man and in individual animals”) of the first chapter, Brodmann explains (Brodmann 1909; Garey 2006):

The comprehensive literature is cited in my earlier works; I shall only mention here those authors who have worked independently in this field; they are: Meynert, Betz, Mierzejewsky, Baillarger, Major, Bevan Lewis, Clarke, Arndt, Berliner, Hammarberg, Roncoroni, Nissl, Kolmer, Bolton, Schlapp, Cajal, Farrar, Koppen, Hermanides, Löwenstein, Campbell, O. Vogt, Mott, Watson, Elliot Smith, Rosenberg, Haller etc.

### Envoi

In an attempt to “rectify certain undeserved historical neglects” of landmark discoveries in cortical cytoarchitectonics, previous papers have revisited the work of individuals who, prior to Brodmann, investigated the histology of the human cerebral cortex (Triarhou 2020, 2021). Herbert Major should be added to this list since, as shown here, he had described and illustrated hexalaminar cortical structure and noted “giant nerve cells” as early as 1875. A case has also been presented suggesting he described and illustrated what later came to be called von Economo neurones in his thesis (Larner and Triarhou 2024). These findings were almost entirely neglected by the neuroscientific community at the time and thereafter. The reasons for this neglect are unclear, but might perhaps relate in part to Major’s key findings being presented in a thesis (Major 1875a) and in a relatively obscure journal (Major 1876) and hence not widely disseminated and available to other researchers. Major’s work only came to our attention as part of ongoing studies of the history of the West Riding Asylum (Larner 2023a, b, 2024).

## Data Availability

Enquiries about data availability should be directed to the authors.
